# Mini-drone assisted tree canopy sampling: a low-cost and high-precision solution

**DOI:** 10.3389/fpls.2023.1272418

**Published:** 2023-10-16

**Authors:** Zhi Liu, Yuanyuan Yan, Jiayin Pang, Qi Guo, Junze Guan, Jiacun Gu

**Affiliations:** ^1^ Key Laboratory of Sustainable Forest Ecosystem Management-Ministry of Education, School of Forestry, Northeast Forestry University, Harbin, China; ^2^ The UWA Institute of Agriculture and School of Agriculture and Environment, The University of Western Australia, Perth, WA, Australia; ^3^ Heilongjiang Institute of Forestry Science, Heilongjiang Academy of Forestry Science, Harbin, China

**Keywords:** branches, canopy sampling, chain saw, foliar sampling, leaves, mini-drone, UAV, wire saw

## Abstract

The collection of tree canopy samples in forest ecosystems has been challenging for researchers and managers during the past decades. Various methods, including pole pruner, tree climber, shooter, throw-line launcher, hydraulic lift (e.g., tower crane) and UAV (unmanned aerial vehicle)-based devices, have been used, however, they are limited by sampling height restrictions, safety hazards to a climber, low retrieving accuracy, high equipment costs, and transportation inconvenience. This study proposed a novel method for collecting tree canopy samples using a portable mini-drone. The mini-drone is operated to pull a traction line across the target branch, drag the retrieving rope to the selected cutting point of the branch, and carry the equipped wire saw or chain saw to cut the canopy sample off. Through on-site testing and field trials, this method was feasible for lower- and middle-canopy sampling (up to 30 meters tall) across most temperate broad-leaved and coniferous tree species. This technique would have great potential in plantation and old-growth forests. Adopting this low-cost mini-drone technique, researchers can collect tree canopy samples safely and efficiently, leading to improvements in relevant physiological and ecological studies focusing on functional traits of branches, leaves, and seeds.

## 1 Introduction

The branches, leaves and seeds are vital organs of trees, frequently sampled and used in research on tree physiology and forest ecology, including hydraulic safety (Bittencourt et al., 2022), photosynthesis and transpiration (Aparecido et al., 2016; Miyata and Kohyama, 2016; Hernández et al., 2020), nutrient resorption (Yu et al., 2022), as well as seed traits. However, collecting tree canopy samples can be challenging in practice.

Traditionally, pole pruner is commonly used to collect branch and leaf samples (Kamoske et al., 2021). However, this method has limitations for tall trees (e.g., over 10 meters), making tree climber an alternative (Anderson et al., 2015). The method of tree climber had safety issues and high labor costs, and some trees or species (e.g., bamboo) are too slim to meet the climbing criteria. To overcome these challenges, researchers have developed a series of conventional techniques and methods over the past decades (Charron et al., 2020), such as hydraulic lift (e.g., tower crane) (Stork, 2007; Gottsberger, 2017), shooter (Gara et al., 2019), or throw-line launcher (Youngentob et al., 2016). However, these methods also have some constraints, including complex operation techniques, inconvenient field transportation, and high equipment costs (Cannon et al., 2021); see detail summary in **Table 1**). Therefore, there is an urgent need for a simple and efficient approach to collect tree canopy samples.

**Table 1:** Summary of canopy sampling methods from tall trees.

Collection methods	Safety	Portability	Precision	Cost	License requirements	Feasibility of trees with height range of 0-10 m	Feasibility of trees with height range of 10-40 m	Canopy shuttle
Pole pruner	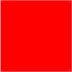	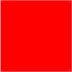	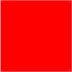	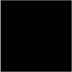	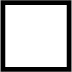	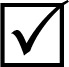	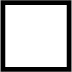	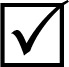
Tree climber	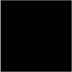	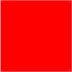	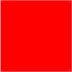	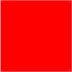	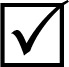	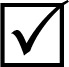	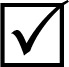	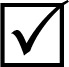
Hydraulic lift (tower crane)	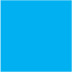	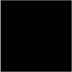	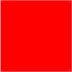	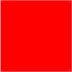	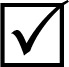	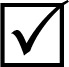	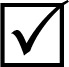	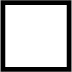
Shooter	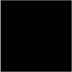	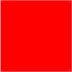	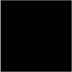	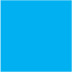	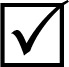	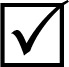	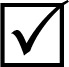	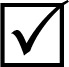
Throw-line launcher	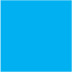	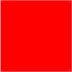	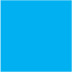	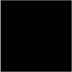	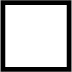	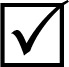	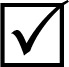	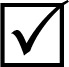
Unmanned aerial vehicle	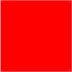	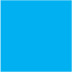	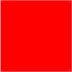	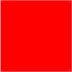	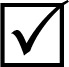	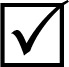	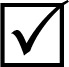	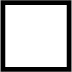
Mini-drone	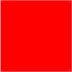	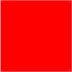	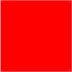	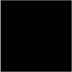	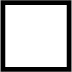 ^**^	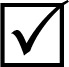	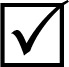	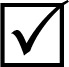

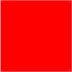
: High. 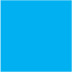 Medium. 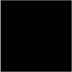;: Low. 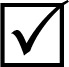: Yes. 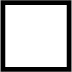: No. ^**^: Mine-drone is under air traffic control in some countries.

In recent years, unmanned aerial vehicle (UAV) technique has become increasingly popular in forestry research and practice, such as forest pest and disease monitoring (Hall et al., 2016; Yuan and Hu, 2016), forest fire monitoring (González-Jorge et al., 2017), wildlife monitoring and identification (Su et al., 2018; Dash et al., 2019), forest resource investigation (Ota et al., 2017), as well as canopy sampling (Charron et al., 2020; La Vigne et al., 2021; Krisanski et al., 2022). As reviewed by Charron et al. (2020), current UAV-based canopy sampling systems can be categorized into two design schemes based on the sampler configuration (lateral-reaching vs. downward-reaching) and cutting mechanism (shear vs. saw). The lateral-reaching sampler configuration is equipped with a shear, while the downward-reaching system is equipped with a saw, both of which enable efficient collection of outermost or uppermost branches of the canopy. However, UAV-based canopy sampling methods have several limitations due to the large size of aircraft system, including the need for professional set-up procedure, skilled operation, working licenses, and large takeoff and landing areas, as well as difficulty in shuttling between tree canopies. Moreover, the UAVs generate high levels of noise that may disturb canopy-dwelling organisms such as bees and birds (Junda et al., 2015; Cannon et al., 2021).

To our knowledge, there was no research exploring the use of mini-drones for tree canopy sampling. We proposed a novel method for collecting branch and leaf samples from tree canopies using a portable mini-drone, in combination with a chain saw and wire saw. The sampling procedure involves controlling the mini-drone to fly over the target branch with a traction line attached. The traction line then pulls a retrieving rope, which is equipped with a wire saw and chainsaw, across the target branch. The saw is used to cut the target branch, allowing for the collection of branch or leaf sample. The method is simple to conduct, accurately captures the target branch to avoid randomness and potential damage to trees, and overcomes limitations of other methods such as the inability to obtain samples from slim and tall trees. In addition, the materials and equipment used in this method are easy to obtain, low cost, and simple to assemble, making the entire collection system portable and suitable for repetitive sampling in the field. We provided a detailed description of the operation procedure and an instructional video tutorial on basic equipment set-up, operation, and additional techniques in **Supplementary Video S1**.

## 2 Materials and methods

### 2.1 Materials and custom-made equipment

**Figure 1:**
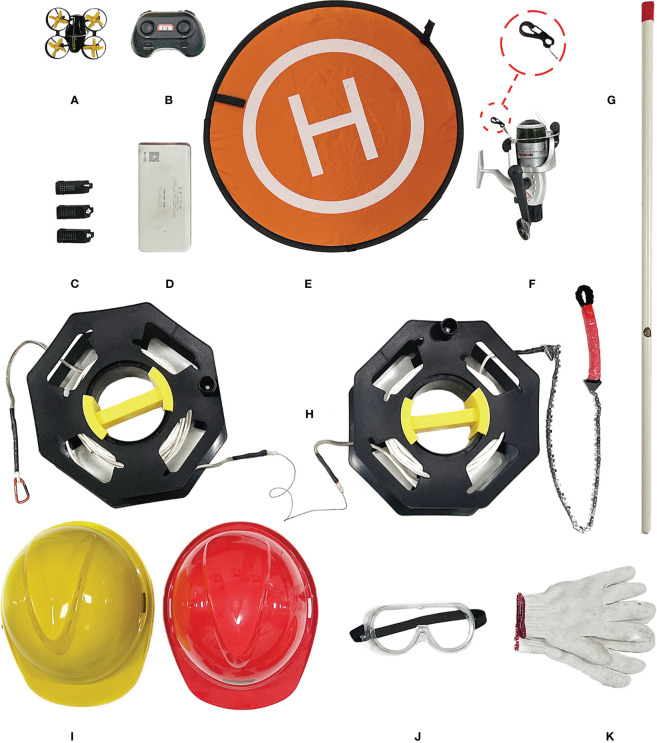
Basic equipment for collecting samples including **(A)** mini-drone, **(B)** mini-drone remote controller, **(C)** mini-drone battery, **(D)** portable charger, **(E)** foldable drone landing pad, **(F)** traction line and spinning fishing reel (local amplification indicates the metal mini-spring buckle), **(G)** guide tube for traction line, **(H)** retrieving rope, which is coiled by two extension cord storage reels, **(I)** safety helmet, **(J)** safety glasses, and **(K)** knit cotton gloves.

The equipment described in this paper were measured in metric units (**Table S1**). The mini-drone used for this study was highly portable and compact, without an obstacle avoidance function but with a flashing light, which facilitates maneuvering through the tree canopy and locating the drone (**Figures 1A, B**). Ten mini-drone batteries (**Figure 1C**), one portable charger (**Figure 1D**), and one foldable drone landing pad (**Figure 1E**) were used.

In addition, several custom-made equipment were also developed, including: (1) a traction line system (**Figure 1F**), consisting of fishing line, mini-spring buckle, and spinning fishing reel; (2) a guide tube for the traction line (**Figure 1G**); (3) a retrieving rope (**Figure 1H**), composed of nylon rope, wire saw, and chain saw. For safety purposes, the safety equipment were used including a safety helmet (**Figure 1I**), safety glasses (**Figure 1J**) and knit cotton gloves (**Figure 1K**). The total cost for the entire equipment kit was approximately US$118.

### 2.2 Setting up and usage of custom-made equipment

The following is specific description and usage guide for the custom-made equipment.

(1) Guide tube for traction line (**Figure S1**): The main component is a one-meter-long PVC pipe. Drill a 30 mm diameter hole at 300 mm from the bottom end of the tube to allow the mini-spring buckle of the traction line to pass through. The guide tube may reduce the risk of the traction line getting caught by the understory vegetation during takeoff of the mini-drone. In addition, the guide tube can extend the buffer area when the mini-drone is hovering for adjustment, ensuring a successful flight.

(2) Traction line (**Figure 1F**): Connect one end of the fishing line to the mini-spring buckle and securely seal it with hot melt adhesive. Wrap the remaining line around the spinning fishing reel for subsequent releasing and retrieving. As the pulling force of the mini-drone is limited, the retrieving rope cannot be directly pulled to the position of target branch. Therefore, operators use mini-drone to take the traction line across the target branch, then connect it to the retrieving rope to pull it to the final position.

(3) Retrieving rope (**Figure S2**): First, prepare two lightweight and strong nylon ropes. The length of the nylon rope is jointly determined by the height of target branch and cutting angle. For example, if the target branch is 30 meters high, and the cutting angle is 60°, then the length of each rope should be 52 meters. As shown in **Figure S2**, for one end of a nylon rope is labelled “I”, equipped with a detachable stainless steel carabiner, while the other end is labelled as “II”. The other nylon rope’s ends are labelled as “III” and “IV”. Second, attach red wear tape to the rope ends of the “II” and “III”, pass them through each end of the wire saw, form a “V” shape, sew them tightly with cotton thread, and fasten the rope ends with a 40 mm long heat shrink tube (**Figure S3A**). To reduce friction on the nylon rope, fill the gap between shirk tubes and ropes with hot melt adhesive (**Figure S3B**). Finally, connect the end “IV” of the nylon rope to the chain saw and leave a ring at the end “V” of the chain saw to connect the stainless steel carabiner of the end “I” of the nylon rope, then tighten it with a 40 mm long heat shrink tube (**Figure S3C**). The retrieving rope has a wire saw and chain saw, which enable it to flexibly cut target canopy samples of different sizes.

### 2.3 Field sampling

#### 2.3.1 Criteria for sampling tree, stand and species

The selection of target branches and affiliated leaves or seeds requires careful consideration of the following key factors.

One important consideration is the range of sampled tree height, which should be no more than 30 meters to ensure the mini-drone can safely shuttle within and between tree canopies. To prevent the mini-drone from getting out of control, it is also essential to maintain a safe distance of 20 meters. Therefore, the mini-drone chosen for this study has a maximum flight range of 50 meters.

Another critical factor to consider is the sampling species and stand. Tree species with clear branching and low stem density are ideal for this method. Open-crowned and sparsely branches species, e.g., those broad-leaved species from *Fraxinus*, *Betula*, *Quercus*, *Acer*, and conifer species such as *Larix*, *Pinus*, and *Abies*, are suitable for sampling. We also expect such method can be applied in most *Eucalyptus* species. However, the current method may not be applicable to dense-branched and high-density species such as spruce. The stem density that target trees sampled should not be too high, the actual density for the species examined in this study was less than 2000 ha^-1^. Therefore, this method is more suitable for plantations with low stem density, or old growth with sparse canopy.

**Figure 2:**
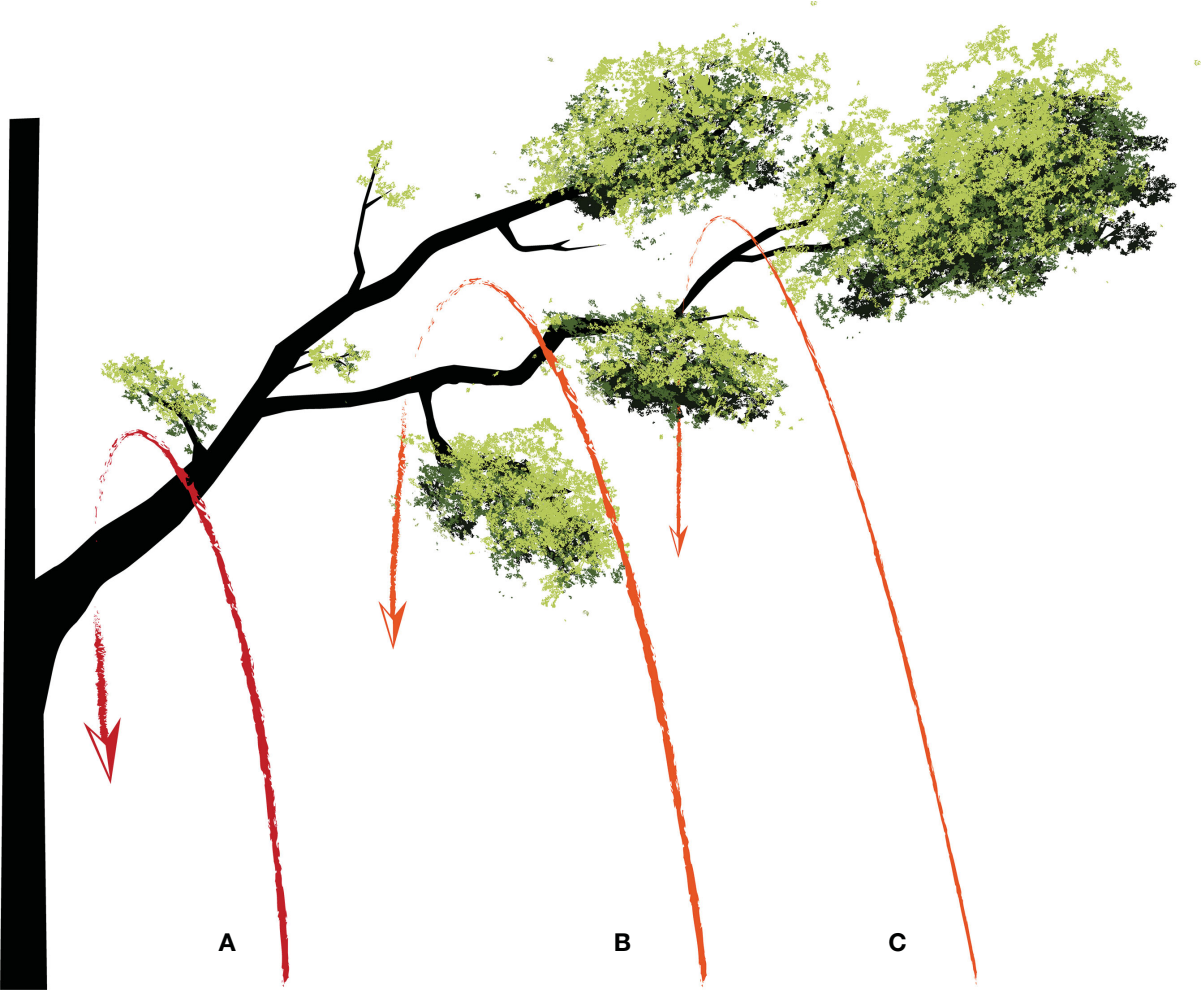
Schematic diagram showing three typical flight paths of a mini-drone for retrieving **(A)** whole branch and leaves, **(B)** small twigs and leaves, and **(C)** only leaves.

In addition, other factors such as selection of target branch, flight path, cutting point and saw type should be considered as well. The selection of target branch should be based on the research proposal. The appropriate flight path and cutting point should be based on the branch size and expected samples (e.g., whole branch or only part leaves). For hydraulic study, flight path “a” (**Figure 2A**) is recommended to ensure a relatively complete branch following the cut. For nutrient resorption study, only small twigs and leaves are needed, so flight path “b” (**Figure 2B**) can be chosen. For the study of photosynthesis and transpiration rate, only leaves are needed, so flight path “c” (**Figure 2C**) is more suitable. It is important to choose the right chain saw or wire saw based on the branch diameter. The diameter of the target branch should be estimated, if necessary, a telescope could be used. A chain saw should be used if the diameter of the branch is greater than 30 mm (**Figure S4A**), while a wire saw should be an appropriate choice if the diameter indicates weak supporting strength (**Figure S4B**). To prevent accidents, ensure that there are no other branches underneath the target branch so that it can drop directly to the ground following the cut. Finally, it should be noted that the current method does not have strict requirements on the branching angle of the target branch to the trunk (e.g., > 45°, Youngentob et al., 2016).

#### 2.3.2 Retrieving procedure

The canopy sampling procedures involves at least two operators collaborating with each other. Operator 1 (referred to as “OT1”) is responsible for controlling the mini-drone (**Figures 1A, B**), while Operator 2 (“OT2”) controls the finishing reel (**Figure 1F**) and guide tube (**Figure 1G**) to release the traction line. They work together to cut off the target branch.

The retrieving procedure involves four steps. The detailed procedure could be better understood by watching **Supplementary Video S1**.

**Figure 3:**
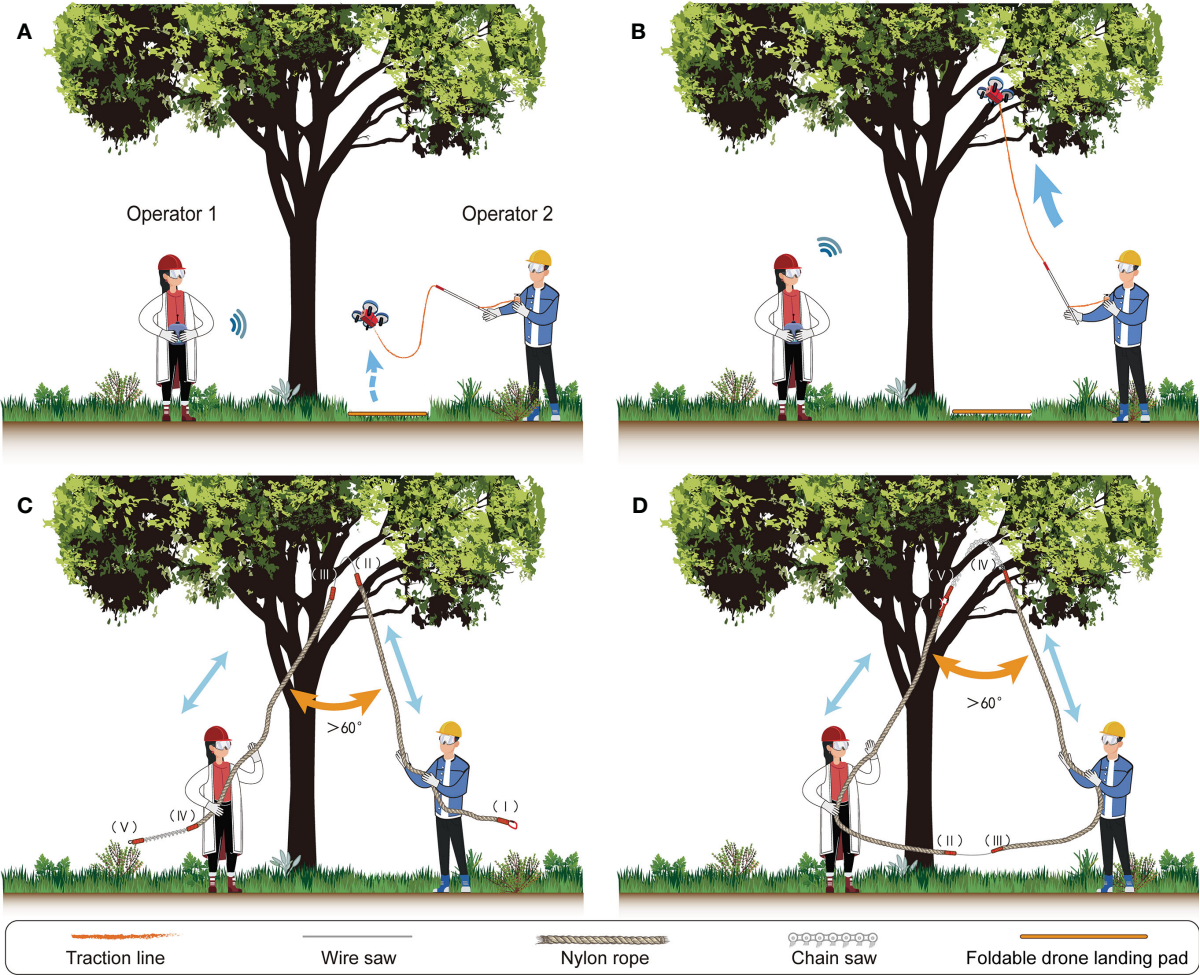
Collecting procedure for operator 1 (OT1) and operator 2 (OT2) in the field. **(A)** OT1 hovers the mini-drone over the understory while OT2 releases the traction line. **(B)** OT1 flies the mini-drone over the target branch. **(C)** OT1 and OT2 cut the target branch with a wire saw. **(D)** OT1 and OT2 cut the target branch with a chain saw.

First, OT1 selects a relatively flat area and clear away any shrubs or tall herbs, before laying down the foldable drone landing pad on the ground. Meanwhile, OT2 sets up the traction line with the mini-spring buckle, attaching it to the inlet of the guide tube and taking it out of the outlet (**Figure S1**). They should then attach the traction line to the mini-drone via the mini-spring buckle (**Figure S5**), and place the mini-drone on the landing pad for takeoff. Subsequently, OT2 adjusts the guide tube and spinning fishing reel, preparing for the release of the traction line. When the mini-drone operated by OT1 takes off and hovers at *c*. 1.3 meters for flight adjustment, OT2 cooperatively releases the traction line (**Figure 3A**). In order to reduce friction between the traction line and the outlet of the guide tube, OT2 should adjust the angle of the guide tube to follow the flight direction of the mini-drone (**Figure 3B**).

**Figure 4:**
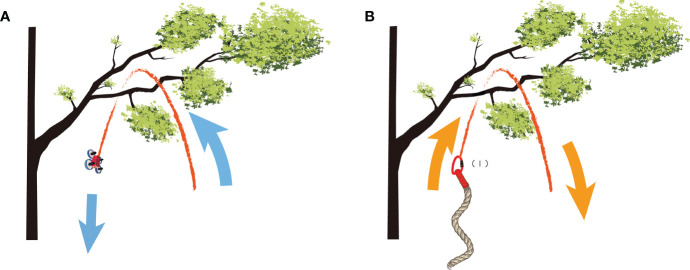
Schematic diagram illustrating the retrieval of branches and leaves using a mini-drone, traction line, and retrieving rope. **(A)** The mini-drone pulls the traction line through the branches. **(B)** The traction line pulls the retrieving rope across the target branch.

Second, OT1 continuously flies the mini-drone with the traction line over the target branch, being careful not to let the traction line touch the bark or twigs of the target tree, as this can cause the mini-drone to become unbalanced. When mini-drone passes through the target branch, OT1 should temporarily descend the mini-drone for a while and turn off the motor of drone. Then, OT1 can use the gravity of the mini-drone to pull the traction line while passing through the target branch (**Figure 4A**). Meanwhile, OT2 continues releasing the traction line until the mini-drone reaches the ground. Ultimately, the traction line forms an “n” shape across the target branch (**Figure 4A**).

Third, OT1 removes the mini-spring buckle from the mini-drone and attaches it to the end “I” of the retrieving rope (**Figure 4B**). OT2 pulls the retrieving rope until the wire saw reaches the cutting point on the target branch (**Figure 3C**). If the diameter of the target branch is more than 30 mm, OT2 should connect the end “I” of the retrieving rope to the “V” end using a stainless steel carabiner (**Figure 3D**). OT1 then pulls the retrieving rope until the chain saw is in position to start cutting the cutting point. The retrieving rope forms an “n” shape across the target branch at this point (**Figure 3D**).

Last, two operators alternately pull the retrieving rope back and forth, and cut off the target branch. Branch and leaf samples then can be collected successfully and processed for further analysis.

To improve the success rate and safety of the collection process, the following key points should be taken into consideration.

(1) Safety should always be the top priority. Operators must wear safety glasses, helmet and cotton glove (**Figure 1**), and ensure that no people or tools are beneath the target branch to prevent potential injury from fallen samples.
(2) OT1 should practice drone control skills of drone, such as hovering adjustment, to ensure that the mini-drone can pass through the target branches successfully.
(3) To simplify the operation, operators should plan the flight path in advance and choose a location with good visibility as the take-off point.
(4) To minimize the risk of maloperation, OT1 and OT2 should practice together multiple times to improve their cooperative proficiency.
(5) If there are dead or obstacle branches under the target branch that prevents the mini-drone’s flight or impede drop of the target branch, operators could remove the obstacle branches before collection using the current method.
(6) When using a wire saw or chain saw to cut the branch, the cutting angle of the retrieving rope should be greater than 60° (**Figures 3C, D**), and the direction of the saw blade should be adjusted to be as perpendicular to the branch as possible to avoid difficulties or the saw getting stuck.
(7) Based on our filed experience, preparing 10 mini-drone batteries (**Figure 1C**) can support about 8 h collection. One battery can support two flights, with each flight time lasting about three minutes. A portable charger (**Figure 1D**) can charge two mini-drone batteries concurrently, and it takes 30 minutes to fully charge.
(8) For safety and efficiency, try to fly the mini-drone on sunny and windless days.

## 3 Discussion

### 3.1 Solutions to unexpected accidents

During the collection process, unexpected accidents may happen, and we proposed several specific solutions to address them.

(1) If the mini-drone gets stuck on a branch or twig, operators can cut the fishing line and connect it to a mini-spring buckle to make a new traction line. A spare mini-drone can then be used to fly again, cut off the branch that the previous mini-drone was stuck on, and finally recover the mini-drone on the ground. We recommend bringing three mini-drones for each field trip.

(2) If the rough branch bark (e.g., some conifer species) prevents the mini-spring buckle from passing over (**Figure 4B**), operators can pull back the retrieving rope to the ground, temporarily wrap the mini-spring buckle with smooth tape to reduce friction, and then try to pass the retrieving rope across the target branch again.

### 3.2 Improvement potential

While the current method efficiently collects canopy samples, there are still some areas for improvement. Firstly, if the cost of the mini-drone is not a limiting factor, a more powerful motor should be selected to increase the flight distance and height, thereby expanding the range of sample tree height that can be collected using this method. Secondly, a mini-drone with a long hovering time, strong self-balance function, and higher-resolution camera should be preferred. This will enable operators to directly identify and select the target branch, improving collection efficiency.

## 4 Conclusions

The proposed method for collecting canopy samples from tall trees using a mini-drone is efficient, accurate and convenient, with no need for an extra working permit. It is suitable for sampling broad-leaved species such as *Fraxinus*, *Betula*, *Quercus*, *Acer*, *Eucalyptus*, and conifer species such as *Larix*, *Pinus*, and *Abies*, as well as bamboos. The method can be used in plantations with low stem density or old-growth forests with sparse canopy. Overall, researchers in tree physiology and forest ecology can collect canopy samples efficiently and safely at a low cost using this method.

## Data availability statement

The original contributions presented in the study are included in the article/**Supplementary Material**. Further inquiries can be directed to the corresponding author.

## Author contributions

ZL: Writing – original draft, Writing – review & editing, Methodology, Investigation. YYY: Writing – original draft, Methodology. JYP: Methodology, Writing – original draft, Writing – review & editing. QG: Writing – review & editing, Methodology, Investigation. JZG: Writing – review & editing, Investigation, Methodology. JCG: Writing – review & editing, Methodology, Conceptualization, Project administration, Supervision, Writing – original draft.
