# Supplementary material for: Mini-drone assisted tree canopy sampling: a low-cost and high-precision solution
Source: Front Plant Sci. 2023 Oct 16;14:1272418. doi: 10.3389/fpls.2023.1272418 (PMC10622779; doi:10.3389/fpls.2023.1272418)
Supplement: Supplementary file 1 [file DataSheet_1.docx]

Supplementary Material

**Article title:**

A novel method of a low-cost, precise collection of leaves and branches from tall trees by using mini-drone

Authors: Zhi Liu, Yuanyuan Yan, Jiayin Pang, Junze Guan, Qi Guo, Jiacun Gu*

Article acceptance date:

The following Supporting Information is available for this article:

**Supplementary Table S1** Key parameter and size in metric unit for devices that used in this study.

**Supplementary Figure S1.** Guide tube for traction line. Please note the outlet (O) and inlet (I). Arrows indicate the direction of the traction line passing through the guide tube.

**Supplementary Figure S2.** Set-up of the retrieving rope. (a) Schematic diagram. (b) Physical picture. The interval between I and II is nylon rope, between II and III is wire saw, between III and IV is nylon rope, and between IV and V is a chain saw. There are two extension cord storage reels for coiling ropes.

**Supplementary Figure S3** Custom-made details for connecting wire and chain saws to the nylon rope of the retrieving rope. The nylon rope is connected to the wire saw without heat shrink tube (a), with heat shrink tube (b), and to the chain saw (c).

**Supplementary Figure S4** Snapshot for cutting branches by using (a) a chain saw and (b) a wire saw.

**Supplementary Figure S5** Custom-made details for connecting mini-drone to mini-spring buckle. The green wire is fishline. Please note that the knot on the buckle is protected by hot melt adhesive.

**Supplementary Video S1** A video tutorial on basic equipment set-up, operation, and additional techniques for canopy sampling using a mini-drone.

**Supplementary Table S1** Key parameter and size in metric unit for devices that used in this study

| **Number** | **Devices** | **Key parameters** | **Dimensions** | **Manufacturer in China** |
| --- | --- | --- | --- | --- |
| 1 | Mini-drone | Maximum flight distance: 50 m | 78 × 90 × 35 mm | Lingke technology corporation |
| 2 | Mini-drone battery | Maximum capacity: 200 mA | 50 × 20 × 15 mm | Lingke technology corporation |
| 3 | Foldable drone landing pad | Texture of material: oxford | 750 × 750 mm | Starirc company |
| 4 | Portable charger | Maximum capacity: 20000 mA | 167 × 80 × 22.6 mm | Romoss company |
| 5 | Fishing line | Net tension: 22.6 kg | 80 m | Qiansifang fishing tackle company |
| 6 | Mini-spring buckle | Texture of material: 304 stainless-steel | 25 × 4 mm | The wolf spirit company |
| 7 | Spinning fishing reel | Maximum bearing capacity: 200 m | 150 × 140 × 140 mm | Guangwei company |
| 8 | Guide tube for traction line | Texture of material: Polyvinyl chloride | 1 m | Rifeng company |
| 9 | Nylon rope | Tensile strength: 127 kg | 100 m | Youran company |
| 10 | Heat shrink tube | Texture of material: Polyolefins | 10 mm | Elecall company |
| 11 | Wire saw | Saw diameter: 1.68 mm | 530 mm | Okoutdoor company |
| 12 | Chain saw | Sawtooth specification: 33 teeth | 650 mm | Okoutdoor company |
| 13 | Extension cord storage reel | Maximum bearing capacity: 212 m | 330 × 330 × 95 mm | Cnoble company |
| 14 | Safety glasses | Texture of material: Polycarbonate | XL | Hengrun Labor protection appliance company |
| 15 | Knit Gloves | Texture of material: cotton | XL | Mingxing company |
| 16 | Safety helmet | Maximum mechanical impact: 2292 N | XL | Likai company |


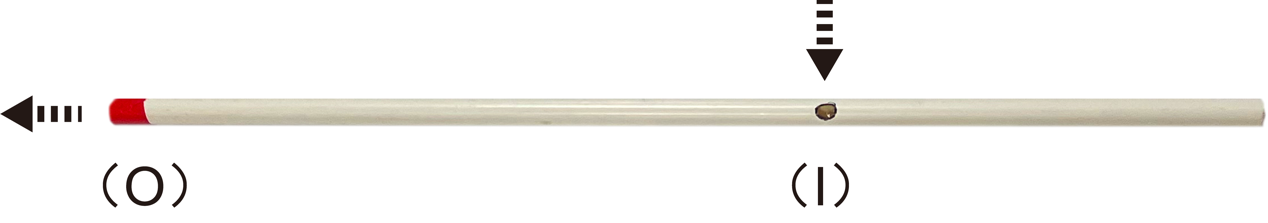


**Supplementary Figure S1** Guide tube for traction line, with the outlet (O) and inlet (I) labelled. Arrows indicate the direction of the traction line passing through the guide tube.


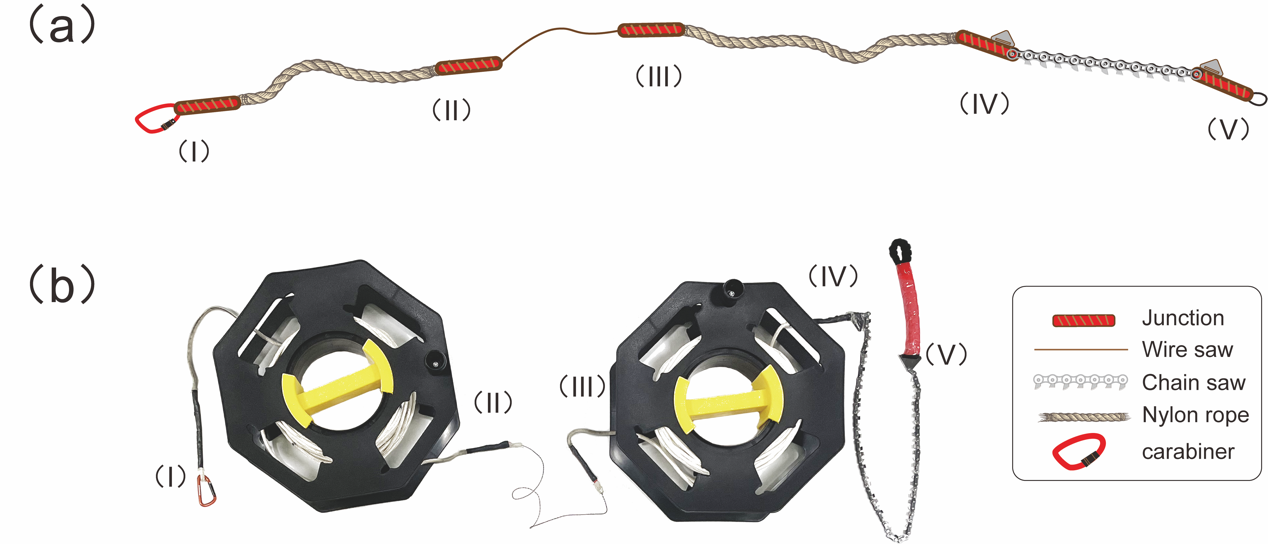


**Supplementary Figure S2** Set-up of the retrieving rope. (a) a schematic diagram and (b) a physical picture. The interval between I and II is nylon rope, between II and III is a wire saw, between III and IV is nylon rope, between IV and V is a chain saw. Two extension cord storage reels are used to coil the ropes.


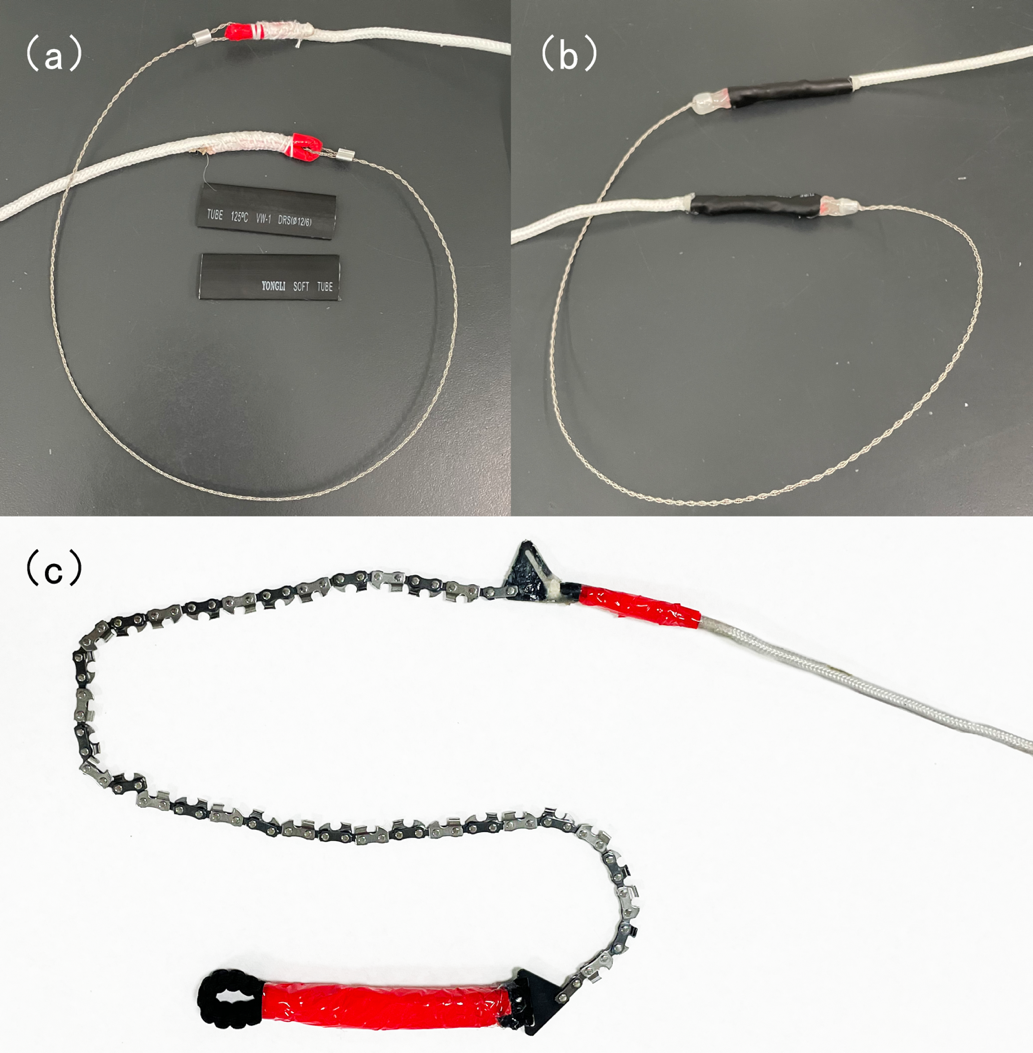


**Supplementary Figure S3** Custom-made details for connecting wire and chain saws to the nylon rope of the retrieving rope. The nylon rope is connected to the wire saw without heat shrink tube (a), with heat shrink tube (b), and to the chain saw (c).


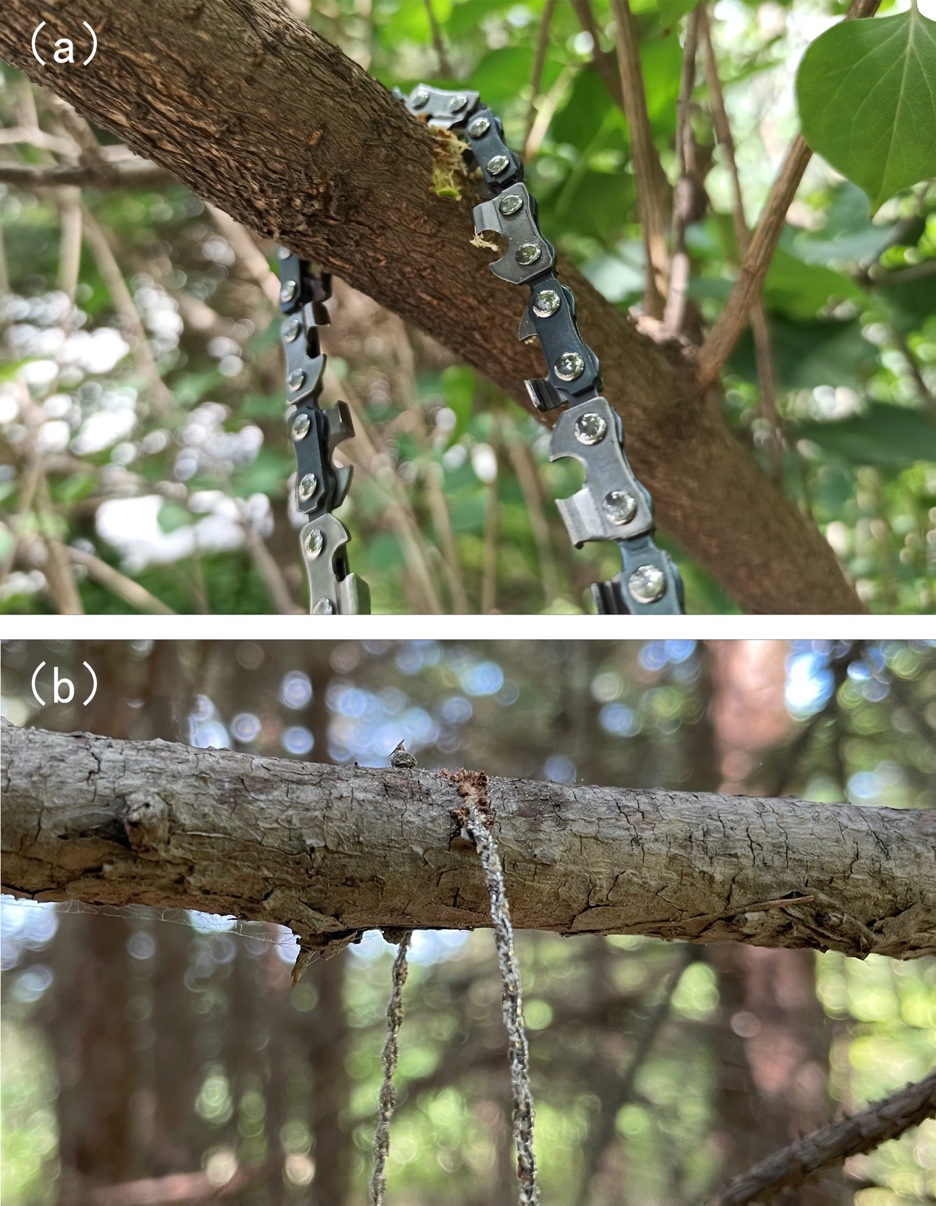


**Supplementary Figure S4** Snapshots showing the process of cutting branches using (a) a chain saw and (b) a wire saw.


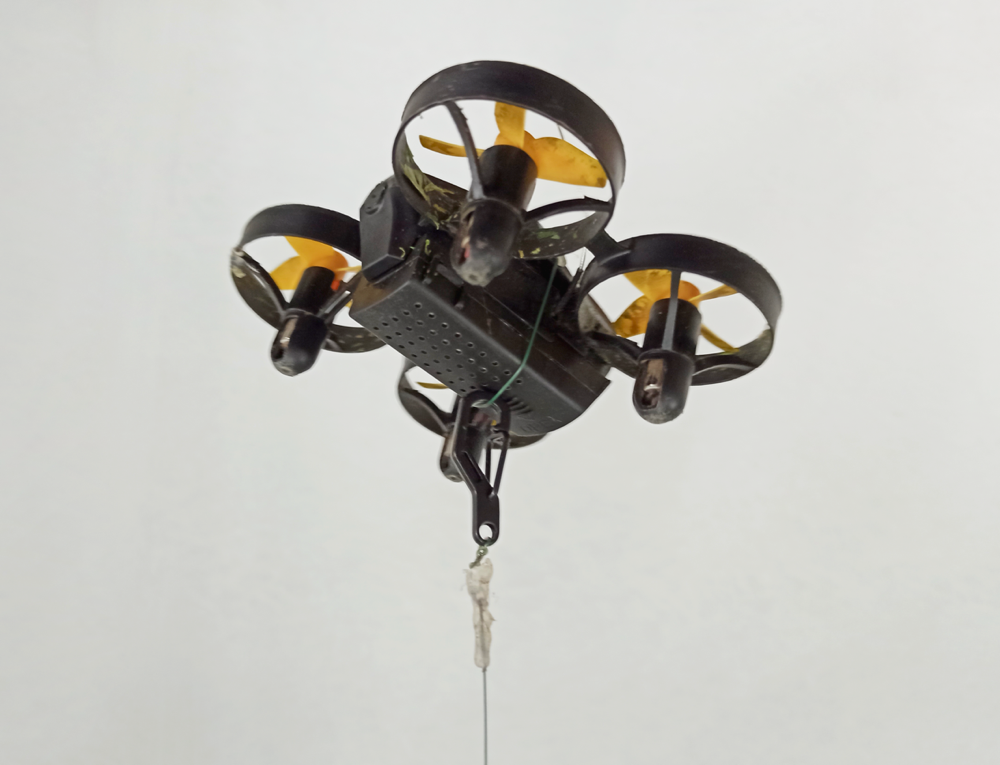


**Supplementary Figure S5** Custom-made details for connecting a mini-drone to a mini-spring buckle. The green wire is fishline, and the knot on the buckle is protected by hot melt adhesive.
